# Clinical, Laboratory, Infectious, and Intervention Factors Associated with ICU Mortality: A Retrospective Cohort Study

**DOI:** 10.3390/jcm15145452

**Published:** 2026-07-12

**Authors:** Mateusz Bartoszewicz, Samuel Stróż, Sławomir Lech Czaban, Jerzy Robert Ładny

**Affiliations:** 1 Department of Anaesthesiology and Intensive Care, Medical University of Bialystok, Marii Sklodowskiej-Curie 24A St., 15-276 Bialystok, Poland; 2 Department of Clinical Immunology, Medical University of Bialystok, Marii Sklodowskiej-Curie 24A St., 15-276 Bialystok, Poland; 3 Department of Emergency Medicine, Medical University of Bialystok, Marii Sklodowskiej-Curie 24A St., 15-276 Bialystok, Poland

**Keywords:** intensive care unit, ICU mortality, in-hospital mortality, TISS-28, bloodstream infection, ventilator-associated pneumonia, catheter-associated urinary tract infection, biomarkers, critical care, retrospective cohort, logistic regression

## Abstract

**Background/Objectives**: Intensive care unit (ICU) mortality reflects interactions between baseline vulnerability, acute physiological derangement, ICU-acquired infection, and the intensity of organ-support therapy. **Methods**: This single-center retrospective cohort study included 3323 adult first ICU hospitalizations at the University Clinical Hospital in Bialystok, Poland, between 1 January 2017 and 1 June 2023. Secondary ICU admissions/readmissions, patients aged <18 years, and one pregnancy admission were excluded. Patients were classified as ICU survivors (*n* = 1778) or ICU non-survivors (*n* = 1545). Variables were compared using *t*-tests, chi-square tests, or Fisher exact tests, and an adjusted logistic regression model was fitted as an exploratory prognostic model. **Results**: ICU mortality was 46.5%, and 28-day ICU mortality was 40.2%. Non-survivors were older than survivors (66.7 ± 15.1 vs. 60.9 ± 17.2 years; *p* < 0.001) and more frequently had arterial hypertension, diabetes mellitus, COVID-19, ischemic heart disease, atrial fibrillation, renal failure, and acute myocardial infarction or ischemic stroke. In the adjusted model, ICU mortality was associated with age per 10 years (OR 1.32, 95% CI 1.18–1.47), COVID-19 (OR 3.15, 95% CI 2.07–4.79), ventilator-associated pneumonia (OR 1.68, 95% CI 1.22–2.30), lactate per 1 mmol/L (OR 1.29, 95% CI 1.16–1.43), pH per 0.1-unit decrease (OR 1.79, 95% CI 1.41–2.29), mechanical ventilation (OR 14.74, 95% CI 3.40–63.87), cardiopulmonary resuscitation (OR 9.45, 95% CI 4.67–19.13), renal replacement therapy (OR 2.01, 95% CI 1.39–2.91), and treatment of acidosis or alkalosis (OR 1.95, 95% CI 1.29–2.94). **Conclusions**: ICU non-survival was associated with older age, COVID-19, cardiovascular and renal vulnerability, ICU-acquired infection, inflammatory and metabolic dysfunction, and early requirement for rescue organ-support interventions. These findings should be interpreted as adjusted associations, not causal effects.

## 1. Introduction

The intensive care unit (ICU) is designed to provide continuous monitoring and advanced organ-support therapy for patients with acute, life-threatening physiological instability. Despite improvements in ventilation, hemodynamic support, renal replacement therapy, infection management, and multidisciplinary critical care organization, mortality after ICU admission remains substantial. Outcome after critical illness is rarely determined by a single factor; rather, it reflects the interaction between baseline vulnerability, the severity of the acute insult, the inflammatory and metabolic host response, ICU-acquired complications, and the intensity of therapeutic interventions required to maintain organ function [1].

Baseline physiological reserve is an important determinant of survival in critically ill patients. Older age, diabetes mellitus, cardiovascular disease, atrial fibrillation, chronic kidney disease, and acute cardiovascular events may reduce the capacity to compensate for shock, hypoxemia, sepsis, or postoperative complications. These factors may accelerate progression from isolated organ dysfunction to multi-organ failure and may modify the response to standard ICU interventions [2]. For this reason, descriptive cohort studies that jointly evaluate demographics, comorbidity, laboratory abnormalities, infection, and treatment intensity remain clinically useful.

Inflammatory and metabolic variables measured at ICU admission provide early information about the severity of acute illness. Biomarkers such as interleukin-6 (IL-6), C-reactive protein (CRP), and procalcitonin (PCT) are widely used to assess the magnitude of systemic inflammation and the probability or severity of infection [3]. Arterial lactate, pH, bicarbonate, PaO2, and PaCO2 provide complementary information on tissue perfusion, acid–base balance, oxygenation, and ventilation. Elevated lactate and acidosis are particularly important because they reflect impaired oxygen delivery, mitochondrial stress, or severe circulatory failure and have been repeatedly associated with adverse short-term outcomes in ICU populations [4].

The regional context should also be considered when interpreting ICU mortality. Previous analyses have reported high mortality among ICU patients in Poland, with substantial differences compared with several other European settings [5,6]. Such differences may reflect case mix, admission thresholds, pre-ICU triage, availability of intermediate-care beds, and healthcare-system organization rather than a single measure of ICU quality. Therefore, contemporary local cohorts are needed to describe which clinical and therapeutic variables are most strongly associated with death in a specific health-system context.

In addition to admission variables, the burden of ICU interventions may serve as a practical marker of disease severity and workload. The Therapeutic Intervention Scoring System-28 (TISS-28) summarizes common ICU activities, including monitoring, airway and respiratory support, vasoactive therapy, renal replacement therapy, nutrition, procedures, and cardiopulmonary resuscitation [7,8]. Daily TISS-28 records can be analyzed either as duration variables, reflecting the number of ICU days on which an intervention occurred, or as binary indicators, reflecting whether an intervention was required at least once during the ICU stay. Both forms may carry clinically relevant information, although duration variables require cautious interpretation because patients who survive longer have more opportunity to accumulate routine ICU-care days.

This study aimed to characterize factors associated with ICU mortality in a large single-center cohort of adult ICU patients. Specifically, we compared survivors and non-survivors with respect to demographic characteristics, comorbidities, COVID-19 status, ICU-acquired infections, admission inflammatory and metabolic laboratory markers, and TISS-28-derived indicators of intervention burden. We additionally fitted an adjusted logistic regression model to report effect estimates and confidence intervals while retaining the descriptive and exploratory nature of the study.

## 2. Materials and Methods

### 2.1. Study Design, Setting, and Reporting Framework

This single-center retrospective cohort study was conducted in the ICU of the University Clinical Hospital in Bialystok, Poland. The source population comprised ICU hospitalizations recorded between 1 January 2017 and 1 June 2023. The study was designed as a hospital-record-based analysis of routinely collected clinical data and was reported in accordance with the Strengthening the Reporting of Observational Studies in Epidemiology (STROBE) framework. The unit of analysis was the eligible ICU hospitalization.

### 2.2. Participants, Outcome, and Cohort Flow

The initial screening set comprised 3504 ICU hospitalizations. Secondary ICU admissions/readmissions were excluded to reduce repeated-measure bias and to focus the analysis on the first eligible ICU hospitalization during the study period (*n* = 178 excluded). Two patients aged < 18 years and one pregnancy admission were also excluded. The final analytic cohort therefore comprised 3323 adult, non-pregnant first ICU hospitalizations.

The primary outcome was ICU mortality, defined as death during the ICU stay. All deaths occurred in the ICU before ICU discharge. Patients were classified as ICU survivors (*n* = 1778; 53.5%) or ICU non-survivors (*n* = 1545; 46.5%). A secondary descriptive outcome was 28-day ICU mortality, defined as death within 28 days after ICU admission.

**Figure 1:**
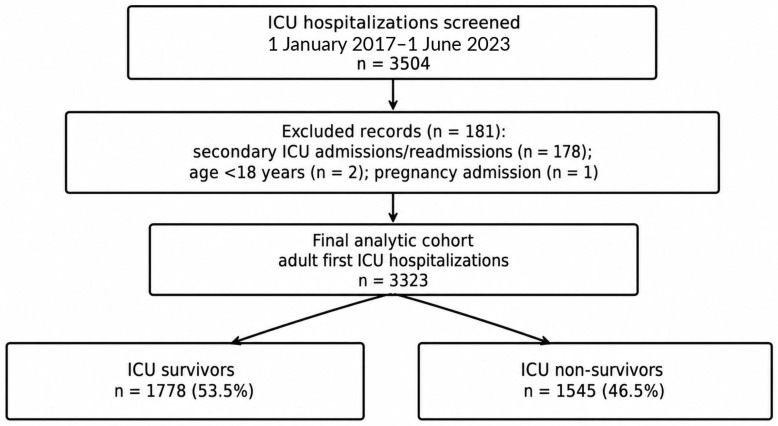
Flowchart of patient screening and inclusion. ICU, intensive care unit.

The patient screening process and outcome classification are summarized in Figure 1.

### 2.3. Data Sources and Clinical Variables

Data were extracted retrospectively from routinely collected hospital and ICU documentation. The analyzed variables were grouped into clinically interpretable domains: demographic characteristics, comorbidities and clinical conditions, ICU-acquired infections, pharmacological treatment variables, ICU length of stay, admission laboratory and gas-exchange variables, and TISS-28 intervention indicators. Demographic variables included sex, age, height, and body weight. Comorbidities and clinical conditions included arterial hypertension, diabetes mellitus, obesity by body mass index (BMI), ischemic heart disease, atrial fibrillation, renal failure, chronic renal failure, heart and/or respiratory failure, acute myocardial infarction or ischemic stroke, and other recorded chronic conditions. Heart failure and respiratory failure could not be separated because the source database stored them as a combined field.

Admission laboratory and gas-exchange variables included CRP, white blood cell count, PCT, IL-6, PaO2, PaCO2, pH, creatinine, glucose, sodium, potassium, total hemoglobin, bicarbonate, and lactate. Admission measurements were defined as values recorded at ICU admission or closest to the initial ICU diagnostic assessment.

### 2.4. Infection and COVID-19 Variables

Infection variables were recorded as binary indicators during the ICU hospitalization. These variables should be interpreted as ICU-acquired or ICU-documented complications and not as baseline predictors. For bacterial bloodstream infection (BSI), positivity required at least four blood samples and at least two positive cultures for a clinically significant organism obtained before the start of antibiotic therapy whenever possible. The microbiological result was evaluated together with the clinical condition of the patient, including fever and laboratory evidence of infection, to reduce misclassification of contamination as true infection. In polymicrobial infections, each causative organism identified in blood culture was considered a unique BSI episode. Bacteremia diagnosed at least 48 h after hospital admission was classified as hospital-acquired BSI. Primary BSI was identified when no definite infection source was documented, whereas secondary BSI was identified when a source was suspected and the same microorganism was detected at the source and in blood culture [9,10].

Ventilator-associated pneumonia (VAP) was defined according to European surveillance principles for ICU-acquired pneumonia. Lower respiratory tract samples were obtained before antibiotic administration when feasible, and the date on which lower respiratory tract samples were positive for suspected VAP pathogens was considered the start of VAP [9]. Catheter-associated urinary tract infection (CA-UTI) required an indwelling urethral catheter for at least 48 h, compatible clinical features such as fever, suprapubic or costovertebral angle discomfort, altered mental status, hypotension, or systemic inflammatory response without another recognized source, and microbiological confirmation. Urine specimens were collected after ICU admission, preferably after catheter replacement to reduce biofilm contamination. A positive culture was defined as ≥10^3^ colony-forming units/mL of one or two bacterial species in a catheter urine specimen; cultures with three or more microorganisms were treated as contaminants and excluded from the CA-UTI definition [11].

Antibiotic treatment was initiated according to clinical judgment, microbiological results, and the presence of fever or other signs of infection. COVID-19 status was based on recorded SARS-CoV-2 diagnostic status during the hospitalization period.

### 2.5. TISS-28 Intervention Variables

TISS-28 items were derived from daily ICU intervention documentation. For each TISS-28 component, two forms of the variable were generated. First, a duration variable was calculated as the number of ICU days on which the intervention was recorded. Second, a binary variable was generated to indicate whether the intervention was recorded at least once during the ICU stay. This approach allowed separation of prolonged exposure to routine ICU care from the occurrence of high-intensity rescue interventions. The analyzed TISS-28 variables included monitoring, laboratory blood sampling, drug administration intensity, dressing care, drain care, respiratory support, artificial airway care, respiratory physiotherapy, vasoactive drug use, fluid-balance interventions, arterial and central venous catheterization, hemodynamic monitoring, cardiopulmonary resuscitation, renal replacement therapy, urine output measurement, forced diuresis, dialysis catheter insertion, treatment of acidosis or alkalosis, parenteral nutrition, enteral nutrition, ICU procedures, procedures outside the ICU, and intracranial pressure monitoring.

### 2.6. Missing Data and Data Quality

The analysis used the available routinely recorded data for each variable. Categorical variables, including comorbidity, infection, pharmacological treatment, COVID-19, mortality outcome, and binary TISS-28 indicators, were complete after data cleaning. Continuous variables had variable missingness, especially height, weight, BMI, and several laboratory or blood-gas measurements. No imputation procedure was applied. For the adjusted logistic regression model, a complete-case approach was used, and the number of included patients is reported with the model results.

Structured APACHE II, SAPS II, and SOFA scores were not available with sufficient completeness and consistency across the whole study period and therefore could not be included. This was treated as an important limitation rather than reconstructed retrospectively from incomplete records.

### 2.7. Statistical Analysis

Continuous variables are presented as mean and standard deviation (SD), and categorical variables are presented as counts and percentages. Between-group comparisons were performed according to ICU mortality status. Continuous variables were compared using Welch *t*-tests. Categorical variables were compared using chi-square tests; Fisher exact tests were used for sparse 2 × 2 tables when expected counts were small. *p*-values are reported consistently as *p*-values. Because many comparisons were performed, *p*-values from univariable tests were interpreted as exploratory and were not used alone to define clinical importance.

The distributions of IL-6, PCT, and lactate were skewed. Therefore, the parametric results for these biomarkers were interpreted cautiously, and Mann–Whitney sensitivity analyses were performed; the direction of association remained unchanged for IL-6, PCT, and lactate. To provide effect estimates and confidence intervals, an adjusted logistic regression model was fitted for ICU mortality. The model was explicitly considered an exploratory prognostic association model, not a causal model. Covariates were selected a priori from clinically relevant domains and included age, sex, major comorbidities, COVID-19 status, infection variables, lactate, pH, creatinine, and selected high-intensity binary TISS-28 interventions. Results are reported as adjusted odds ratios (ORs) with 95% confidence intervals (CIs). A sensitivity model excluding COVID-19 patients was also fitted. Statistical analyses were performed using R software version 4.1.1 [12].

## 3. Results

### 3.1. Cohort Characteristics and Mortality Outcomes

The final analytic cohort included 3323 adult, non-pregnant first ICU hospitalizations. Overall, 1545 patients died during the ICU stay, corresponding to an ICU mortality rate of 46.5%, while 1778 patients survived ICU hospitalization. Because all deaths occurred in the ICU, ICU mortality and in-hospital mortality were identical in the source records. The 28-day ICU mortality rate was 40.2% (1335 deaths within 28 days after ICU admission). COVID-19 was recorded in 355 patients, with 227 deaths among COVID-19 patients (63.9%) compared with 1318 deaths among 2968 non-COVID-19 patients (44.4%).

Table 1 presents baseline characteristics, comorbidities, infection variables, pharmacological treatment variables, admission laboratory and gas-exchange variables, and TISS-28 intervention burden according to ICU mortality.

### 3.2. Demographic and Comorbidity Differences

Non-survivors were older than survivors (66.7 ± 15.1 vs. 60.9 ± 17.2 years; *p* < 0.001). Female sex was slightly more frequent among non-survivors than survivors (40.7% vs. 36.6%; *p* = 0.015), whereas body weight did not differ significantly. Several cardiovascular, renal, metabolic, and infectious clinical conditions were more common among non-survivors. These included arterial hypertension (41.6% vs. 32.7%; *p* < 0.001), diabetes mellitus (20.0% vs. 14.7%; *p* < 0.001), ischemic heart disease (7.4% vs. 4.9%; *p* = 0.003), atrial fibrillation (16.1% vs. 8.7%; *p* < 0.001), renal failure (9.1% vs. 4.5%; *p* < 0.001), and acute myocardial infarction or ischemic stroke (5.2% vs. 3.3%; *p* = 0.007). COVID-19 was also more frequent among non-survivors (14.7% vs. 7.2%; *p* < 0.001).

### 3.3. Infection and Pharmacological Treatment Variables

Bacterial BSI occurred in 374 patients (11.3%) and was more frequent among non-survivors than survivors (13.7% vs. 9.1%; *p* < 0.001). CA-UTI occurred in 396 patients (11.9%) and showed a non-significant trend toward higher frequency among non-survivors (13.0% vs. 11.0%; *p* = 0.076). VAP occurred in 915 patients (27.5%) and was more common among non-survivors (29.4% vs. 25.9%; *p* = 0.025). Dexamethasone and general steroid therapy were recorded less frequently among non-survivors than survivors; these associations should be interpreted cautiously because pharmacological exposure may reflect indication, timing, pandemic period, and survival time.

### 3.4. Admission Laboratory and Gas-Exchange Findings

Admission laboratory values showed a consistent pattern of greater inflammatory and metabolic derangement among non-survivors. Compared with survivors, non-survivors had higher CRP (146.1 ± 109.5 vs. 127.6 ± 89.3 mg/L), white blood cell count (15.9 ± 16.2 vs. 12.9 ± 6.6 10^9/L), PCT (8.8 ± 16.3 vs. 4.9 ± 12.1 ng/mL), and IL-6 (565.8 ± 857.9 vs. 305.9 ± 567.0 pg/mL), with all comparisons reaching *p* < 0.001. Non-survivors also had higher creatinine, glucose, sodium, potassium, lactate, and PaCO2, together with lower pH and bicarbonate. Lactate showed the largest absolute separation between groups (4.4 ± 5.1 vs. 1.8 ± 1.3 mmol/L; *p* < 0.001), and bicarbonate was lower among non-survivors (23.6 ± 6.4 vs. 26.6 ± 4.2 mmol/L; *p* < 0.001). PaO2 and total hemoglobin did not differ significantly between groups.

### 3.5. TISS-28 Intervention Burden

TISS-28 duration variables and binary intervention indicators showed complementary information. Survivors accumulated longer durations of several routine ICU activities, including monitoring, laboratory blood sampling, multiple-drug administration, standard dressing care, artificial airway care, arterial catheterization, central venous catheterization, urine output measurement, forced diuresis, enteral nutrition, and procedures outside the ICU. This pattern most likely reflects survival-time effects and longer opportunity to accumulate routine ICU care days rather than protective effects of these interventions.

By contrast, non-survivors more frequently required high-intensity organ-support and rescue interventions. Mechanical ventilation was recorded at least once in 99.4% of non-survivors compared with 91.5% of survivors (*p* < 0.001). Multiple vasoactive drugs were recorded in 76.2% vs. 52.6% (*p* < 0.001), cardiopulmonary resuscitation in 21.8% vs. 2.2% (*p* < 0.001), renal replacement therapy in 33.7% vs. 12.0% (*p* < 0.001), dialysis catheter insertion in 25.6% vs. 8.7% (*p* < 0.001), and treatment of acidosis or alkalosis in 46.4% vs. 11.6% (*p* < 0.001).

### 3.6. Adjusted Prognostic Model for ICU Mortality

The adjusted logistic regression model included 1278 complete cases, including 616 ICU deaths. The model is presented as an exploratory prognostic association model. After adjustment, ICU mortality remained associated with age, acute myocardial infarction or ischemic stroke, COVID-19, VAP, higher lactate, lower pH, mechanical ventilation, multiple vasoactive drugs, cardiopulmonary resuscitation, renal replacement therapy, and treatment of acidosis or alkalosis (Table 2). BSI and CA-UTI were not independently associated with ICU mortality after adjustment in this complete-case model.

### 3.7. Missing Data

The extent of missing data varied by variable group. Categorical variables, infection indicators, COVID-19 status, pharmacological treatment indicators, and binary TISS-28 indicators were complete after data cleaning. Laboratory and blood-gas values had greater missingness because they depended on the availability of admission-time measurements in routine records. IL-6 had the largest missingness (87.7%), while lactate and blood-gas variables were available for approximately 38.6% of the cohort. No imputation was applied. The extent of missingness is reported in Table 3.

### 3.8. Sensitivity Analysis Excluding COVID-19 Patients

After exclusion of patients with COVID-19, the adjusted model included 986 complete cases and 429 deaths. The overall pattern was consistent with the main model: age, acute myocardial infarction or ischemic stroke, VAP, lactate, lower pH, mechanical ventilation, multiple vasoactive drugs, cardiopulmonary resuscitation, and renal replacement therapy remained associated with ICU mortality. Treatment of acidosis or alkalosis showed the same direction but did not reach conventional statistical significance in this sensitivity model (Table 4).

## 4. Discussion

### 4.1. Principal Findings

This single-center retrospective cohort study provides a detailed description of demographic, clinical, infectious, laboratory, and intervention-related factors associated with ICU mortality among 3323 adult ICU patients. The overall ICU mortality rate was 46.5%, and all deaths occurred during ICU hospitalization. Non-survivors were older, had a higher burden of cardiovascular and renal comorbidity, more frequently had COVID-19 and ICU-acquired infections, and presented with more severe inflammatory, metabolic, and renal dysfunction at ICU admission. They also required high-intensity organ-support interventions more frequently than survivors, particularly mechanical ventilation, multiple vasoactive drugs, cardiopulmonary resuscitation, renal replacement therapy, dialysis catheter insertion, and treatment of acidosis or alkalosis.

The adjusted prognostic model supported a clinically coherent high-risk pattern. Age, acute myocardial infarction or ischemic stroke, COVID-19, VAP, admission hyperlactatemia, acidemia, mechanical ventilation, cardiopulmonary resuscitation, renal replacement therapy, and acid–base rescue therapy remained associated with ICU mortality after adjustment. These results should not be interpreted as proof of causality, because several variables occurred during ICU stay and may represent evolving severity, time-dependent complications, or consequences of treatment indication.

### 4.2. Interpretation of Admission Laboratory Findings

The admission laboratory profile of non-survivors suggests more severe systemic inflammation and impaired tissue perfusion at the beginning of ICU care. Higher IL-6, CRP, PCT, and white blood cell count indicate a stronger inflammatory or infectious burden, while higher lactate, lower pH, and lower bicarbonate indicate metabolic stress and acid–base failure. The mean admission lactate among non-survivors was 4.4 mmol/L, compared with 1.8 mmol/L among survivors. In adjusted modeling, each 1 mmol/L increase in lactate was associated with higher odds of ICU mortality, and each 0.1-unit decrease in pH was also associated with higher odds of death. These findings are consistent with the established interpretation of lactate elevation and acidemia as markers of circulatory failure, impaired oxygen utilization, or severe cellular stress in critical illness [3,4,13,14].

Renal and electrolyte abnormalities also contributed to the non-survivor phenotype. Higher creatinine and potassium at admission, together with more frequent renal replacement therapy and dialysis catheter insertion during the ICU stay, suggest that renal dysfunction was both an early marker of severity and an important component of subsequent organ-support burden. Creatinine was not independently associated with mortality in the complete-case adjusted model after accounting for renal replacement therapy and other severity markers, which likely reflects overlap between baseline renal dysfunction and subsequent renal support.

### 4.3. Infection, COVID-19, Device-Related Infection, and Secondary ICU Complications

BSI, CA-UTI, and VAP were documented during ICU hospitalization rather than solely at baseline. Therefore, these variables should be interpreted as ICU-acquired or ICU-documented complications and as markers of an evolving clinical course. BSI and VAP were more frequent among non-survivors in univariable analyses. In the adjusted complete-case model, VAP remained associated with ICU mortality, whereas BSI and CA-UTI did not. This difference does not exclude clinical importance of BSI or CA-UTI; it may reflect timing, competing risks, survival-time bias, confounding by severity, or overlap with invasive devices and organ-support variables. Time-dependent methods would be required to estimate infection effects more rigorously [15].

Possible sources of BSI in ICU patients include central venous catheters, arterial catheters, dialysis catheters, invasive hemodynamic monitoring devices, urinary catheters, respiratory infections, surgical or wound sources, and other prosthetic or implanted devices. Although the present dataset did not specifically distinguish the source of each BSI or identify cardiac implantable electronic device (CIED) status, device-related infections deserve specific consideration in critically ill cardiovascular patients. CIED infections are associated with substantial morbidity, prolonged antibiotic therapy, device extraction, repeated hospitalization, and mortality. Risk is influenced by patient-related factors such as renal dysfunction, diabetes, frailty, and heart failure, as well as procedure-related factors including generator replacement, pocket hematoma, temporary pacing, and repeated interventions [16,17,18].

Prevention should therefore include careful pre-procedural risk assessment, peri-procedural antibiotic prophylaxis, strict asepsis, meticulous surgical technique, hematoma prevention, and structured post-procedural surveillance. In selected high-risk patients, local antibacterial approaches such as gentamicin-impregnated collagen sponges may be considered, although their use should be guided by institutional protocols, patient risk, and the available evidence [16,17]. Future ICU datasets should capture catheter source, dialysis catheter status, temporary pacing wires, pacemakers, implantable cardioverter-defibrillators (ICDs), cardiac resynchronization therapy devices, and device-pocket infections to better classify BSI origin.

COVID-19 was also more frequent among non-survivors and remained associated with ICU mortality in the adjusted model. The inclusion period overlapped with the pandemic, during which ICU admission patterns, respiratory support strategies, antimicrobial use, infection-control pressures, and resource strain changed substantially. COVID-19 may therefore have influenced mortality directly through viral respiratory failure and indirectly through increased susceptibility to ICU-acquired bacterial infection, prolonged ventilation, and changes in ICU workload [19,20,21,22,23,24]. The sensitivity analysis excluding COVID-19 patients showed broadly consistent associations for the main non-COVID cohort.

### 4.4. Interpretation of TISS-28 Intervention Burden

TISS-28 variables added a clinically useful dimension to the analysis because they captured the intensity and duration of ICU care. A key observation was the difference between routine-care duration variables and high-intensity binary intervention variables. Survivors accumulated more days of several routine interventions, including monitoring, catheter care, airway care, urine output measurement, and nutrition. This does not necessarily mean that those interventions were protective. Rather, it likely reflects survival-time bias: patients who live longer have more time to accumulate routine ICU care days.

For this reason, the revised analysis emphasized binary high-intensity TISS-28 indicators in the adjusted model. Mechanical ventilation, multiple vasoactive drugs, cardiopulmonary resuscitation, renal replacement therapy, and treatment of acidosis or alkalosis were more directly interpretable as markers of severe physiological failure and remained associated with mortality. These findings support the use of TISS-28 not only as a nursing workload instrument but also as a structured marker of intervention burden and disease severity, provided that duration variables are interpreted in light of time at risk and survival time [7,8,25,26].

### 4.5. Post-ICU Cardiovascular Risk Management and Transitional Arrhythmic Risk

Beyond the acute ICU phase, selected patients with recent myocardial injury, cardiomyopathy, cardiac arrest, severe metabolic derangement, or transiently reduced ventricular function may remain at increased risk of malignant ventricular arrhythmias. Some patients may not yet be appropriate candidates for immediate ICD implantation because arrhythmic risk is temporary, recovery of ventricular function is possible, or prognosis and long-term indications require reassessment. In this setting, the wearable cardioverter-defibrillator (WCD) can provide a bridge between ICU discharge or step-down care and definitive ICD decision-making [27,28].

This point is relevant to ICU organization because unnecessary prolongation of ICU stay may increase exposure to nosocomial infection, delirium, deconditioning, catheter-related complications, and resource use. In carefully selected patients, a WCD-based strategy combined with medical optimization, remote monitoring, echocardiographic reassessment, and structured follow-up may support safer transition out of ICU-level care while maintaining temporary protection from sudden cardiac death. This consideration is not a conclusion from the present dataset, which did not contain WCD or ICD indication data, but it provides a practical framework for post-ICU cardiovascular planning in patients recovering from acute critical illness.

### 4.6. Clinical and Organizational Implications

The results have practical implications for ICU risk stratification. A patient presenting with advanced age, cardiovascular or renal comorbidity, high inflammatory markers, hyperlactatemia, acidosis, renal dysfunction, and early need for vasoactive drugs, mechanical ventilation, or renal support should be recognized as having a high-risk profile. Such recognition may support closer monitoring, earlier multidisciplinary review, timely antimicrobial and source-control decisions when infection is suspected, and proactive discussions with patients’ families about prognosis and treatment goals.

The findings may also be useful for resource planning. TISS-28-based data can help quantify the workload associated with different ICU phenotypes. Patients requiring multiple vasoactive agents, renal replacement therapy, and treatment of acid–base disorders typically require greater nursing, medical, and technical resources than patients receiving only routine monitoring and standard care. Combining admission laboratory information with daily intervention-burden data may therefore support more transparent planning of ICU staffing, equipment, and bed utilization.

### 4.7. Limitations

This study has several limitations. First, it was a single-center retrospective analysis, and the results may reflect the case mix, admission thresholds, documentation practices, and resource structure of one tertiary hospital in Poland. Generalizability to other ICUs, regions, or post-pandemic settings may therefore be limited. Second, although an adjusted logistic regression model was added, the analysis remains observational and exploratory. Associations between variables and mortality should not be interpreted as independent causal effects, and confounding by disease severity, treatment indication, time at risk, and survival time remains likely.

Third, structured APACHE II, SAPS II, and SOFA scores were not available with sufficient completeness and consistency across the whole study period. Their absence limits comparison with established prognostic models and may leave residual confounding by severity of illness. Fourth, missing data were substantial for several admission laboratory and gas-exchange variables, especially IL-6 and blood-gas-derived variables. The adjusted model therefore used complete cases and may not fully represent patients without these measurements. Fifth, the study relied on routinely collected clinical documentation, which improves real-world relevance but introduces possible measurement variation, coding variability, and documentation bias.

Sixth, BSI, CA-UTI, VAP, and many TISS-28 interventions were recorded during ICU hospitalization. These variables are time-dependent and may occur after ICU admission, after clinical deterioration, or after initiation of invasive support. Patients who survive longer also have more time to develop ICU-acquired infection or accumulate routine interventions. Future studies should evaluate these variables using time-dependent methods, competing-risk approaches, landmark analyses, or early-versus-late infection stratification. Seventh, the dataset did not identify CIED status, temporary pacing wires, device-pocket infections, exact catheter source, intervention timing, dose, appropriateness, clinical response, or complications related to specific interventions. Finally, the COVID-19 pandemic occurred during the study period and may have affected case mix, mortality, infection rates, and ICU workload. Longer-term post-discharge outcomes were not available.

## 5. Conclusions

In this retrospective cohort of 3323 adult ICU patients, ICU mortality was associated with older age, COVID-19, selected cardiovascular and renal conditions, ICU-acquired infection, severe inflammatory and metabolic abnormalities at ICU admission, and greater requirement for high-intensity rescue interventions. In adjusted prognostic modeling, age, acute myocardial infarction or ischemic stroke, COVID-19, VAP, admission lactate, acidemia, mechanical ventilation, cardiopulmonary resuscitation, renal replacement therapy, multiple vasoactive drugs, and treatment of acidosis or alkalosis were associated with ICU mortality. The combined assessment of admission laboratory markers and TISS-28-derived intervention burden provides a transparent description of the clinical phenotype associated with ICU non-survival. These findings may support early risk recognition, structured ICU outcome reporting, and resource planning, while requiring confirmation in externally validated and time-dependent analyses.

## Figures and Tables

**Table 1:** Baseline characteristics, comorbidities, infections, pharmacological treatment variables, TISS-28 intervention burden, and admission laboratory/gas-exchange variables according to ICU mortality.

Variable	Survivors	Non-Survivors	Total	*p*-Value
Baseline characteristics				
Female sex, *n* (%)	651 (36.6)	629 (40.7)	1280 (38.5)	0.015
Male sex, *n* (%)	1127 (63.4)	916 (59.3)	2043 (61.5)	0.015
Age, years, mean (SD)	60.9 (17.2)	66.7 (15.1)	63.6 (16.5)	<0.001
Height, cm, mean (SD)	168.7 (16.8)	166.9 (15.7)	167.9 (16.3)	0.011
Weight, kg, mean (SD)	80.5 (21.1)	79.9 (21.3)	80.2 (21.2)	0.488
Time from ICU admission to death, days, mean (SD)	Not applicable	13.8 (18.7)	13.8 (18.7) among deaths	—
Comorbidities and clinical conditions				
Arterial hypertension, *n* (%)	582 (32.7)	643 (41.6)	1225 (36.9)	<0.001
COVID-19, *n* (%)	128 (7.2)	227 (14.7)	355 (10.7)	<0.001
Asthma, *n* (%)	34 (1.9)	35 (2.3)	69 (2.1)	0.476
Diabetes mellitus, *n* (%)	261 (14.7)	309 (20.0)	570 (17.2)	<0.001
Obesity by BMI, *n* (%)	342 (19.2)	313 (20.3)	655 (19.7)	0.459
Addison disease, *n* (%)	3 (0.2)	0 (0.0)	3 (0.1)	0.253
Crohn disease, *n* (%)	1 (0.1)	1 (0.1)	2 (0.1)	1.000
Graves–Basedow disease, *n* (%)	2 (0.1)	2 (0.1)	4 (0.1)	1.000
Hashimoto disease, *n* (%)	10 (0.6)	5 (0.3)	15 (0.5)	0.306
Parkinson disease, *n* (%)	16 (0.9)	11 (0.7)	27 (0.8)	0.547
Ischemic heart disease, *n* (%)	88 (4.9)	114 (7.4)	202 (6.1)	0.003
Gastroesophageal reflux disease, *n* (%)	6 (0.3)	5 (0.3)	11 (0.3)	0.945
Peptic ulcer disease, *n* (%)	15 (0.8)	17 (1.1)	32 (1.0)	0.450
Gout, *n* (%)	18 (1.0)	24 (1.6)	42 (1.3)	0.164
Muscular dystrophy, *n* (%)	5 (0.3)	0 (0.0)	5 (0.2)	0.065
Hemophilia, *n* (%)	0 (0.0)	1 (0.1)	1 (0.0)	0.465
Hyperlipidemia, *n* (%)	39 (2.2)	28 (1.8)	67 (2.0)	0.436
Cardiomyopathy, *n* (%)	15 (0.8)	17 (1.1)	32 (1.0)	0.450
Atrial fibrillation, *n* (%)	155 (8.7)	249 (16.1)	404 (12.2)	<0.001
Renal failure, *n* (%)	80 (4.5)	141 (9.1)	221 (6.7)	<0.001
Heart and/or respiratory failure, *n* (%)	703 (39.5)	632 (40.9)	1335 (40.2)	0.423
Acute myocardial infarction or ischemic stroke, *n* (%)	59 (3.3)	80 (5.2)	139 (4.2)	0.008
Acute infectious and/or rheumatic disease, *n* (%)	231 (13.0)	238 (15.4)	469 (14.1)	0.046
Chronic renal failure, *n* (%)	17 (1.0)	13 (0.8)	30 (0.9)	0.727
Thrombophilia, *n* (%)	5 (0.3)	8 (0.5)	13 (0.4)	0.276
Infection variables				
Bloodstream infection, bacterial, *n* (%)	162 (9.1)	212 (13.7)	374 (11.3)	<0.001
Catheter-associated urinary tract infection, *n* (%)	195 (11.0)	201 (13.0)	396 (11.9)	0.070
Ventilator-associated pneumonia, *n* (%)	462 (26.0)	454 (29.4)	916 (27.6)	0.029
Pharmacological treatment variables				
Dexamethasone, *n* (%)	142 (8.0)	73 (4.7)	215 (6.5)	<0.001
Steroid therapy, *n* (%)	549 (30.9)	347 (22.5)	896 (27.0)	<0.001
TISS-28 intervention duration, days, mean (SD)				
Monitoring	18.4 (18.8)	14.3 (17.5)	16.5 (18.3)	<0.001
Laboratory blood sampling	18.3 (18.8)	14.1 (17.5)	16.3 (18.3)	<0.001
One drug administration	0.0 (1.4)	0.0 (0.3)	0.0 (1.0)	0.534
Two-drug administration	2.1 (8.8)	1.8 (8.5)	2.0 (8.7)	0.409
Multiple-drug administration	18.4 (18.8)	14.3 (17.5)	16.4 (18.3)	<0.001
Standard dressing care	16.1 (18.0)	12.3 (16.1)	14.3 (17.2)	<0.001
Frequent dressing changes	0.6 (3.3)	0.4 (2.7)	0.5 (3.0)	0.030
Drain care	4.4 (9.7)	3.4 (10.7)	3.9 (10.2)	0.005
Mechanical ventilation	13.0 (15.0)	13.3 (16.2)	13.2 (15.6)	0.627
HFNC or CPAP respiratory support	2.1 (5.9)	0.4 (2.0)	1.3 (4.6)	<0.001
Respiratory support without mechanical ventilation	8.6 (14.4)	5.5 (14.4)	7.2 (14.5)	<0.001
Artificial airway care	16.5 (19.2)	13.9 (17.3)	15.3 (18.4)	<0.001
Respiratory physiotherapy	11.1 (15.6)	8.7 (12.4)	10.0 (14.2)	<0.001
Single vasoactive drug	7.9 (10.0)	6.6 (10.3)	7.3 (10.2)	<0.001
Multiple vasoactive drugs	4.4 (7.9)	5.9 (9.7)	5.1 (8.8)	<0.001
Massive fluid loss or high fluid administration	1.6 (5.1)	1.3 (4.4)	1.5 (4.8)	0.033
Arterial catheter	17.9 (18.0)	14.0 (16.8)	16.1 (17.6)	<0.001
Pulmonary artery catheter	0.0 (0.1)	0.0 (0.1)	0.0 (0.1)	0.289
Central venous catheter	17.7 (18.3)	14.0 (17.4)	16.0 (18.0)	<0.001
Cardiopulmonary resuscitation	0.0 (0.2)	0.2 (0.5)	0.1 (0.4)	<0.001
Calibrated minimally invasive hemodynamic monitoring	0.3 (1.9)	0.3 (2.4)	0.3 (2.1)	0.390
Non-calibrated minimally invasive hemodynamic monitoring	0.0 (0.1)	0.0 (0.0)	0.0 (0.1)	0.317
Renal replacement therapy	1.4 (5.4)	3.3 (8.0)	2.3 (6.8)	<0.001
Urine output measurement	18.3 (18.7)	14.1 (17.5)	16.4 (18.3)	<0.001
Forced diuresis	14.0 (16.9)	10.4 (15.3)	12.3 (16.3)	<0.001
Dialysis catheter insertion	0.1 (0.4)	0.3 (0.6)	0.2 (0.5)	<0.001
Treatment of acidosis/alkalosis	0.2 (0.7)	1.0 (1.5)	0.6 (1.2)	<0.001
Parenteral nutrition	7.9 (14.1)	6.6 (13.7)	7.3 (14.0)	0.010
Enteral nutrition	13.5 (17.0)	9.0 (14.3)	11.4 (15.9)	<0.001
Single ICU intervention	1.2 (1.7)	1.0 (1.7)	1.1 (1.7)	0.008
Multiple ICU interventions	0.1 (0.4)	0.1 (0.4)	0.1 (0.4)	0.522
Procedures outside the ICU	0.9 (1.8)	0.7 (1.4)	0.8 (1.6)	<0.001
ICP monitoring	0.0 (0.2)	0.0 (0.2)	0.0 (0.2)	0.687
TISS-28 intervention recorded at least once, *n* (%)				
Monitoring	1761 (99.0)	1540 (99.7)	3301 (99.3)	0.025
Laboratory blood sampling	1748 (98.3)	1526 (98.8)	3274 (98.5)	0.275
One drug administration	8 (0.4)	11 (0.7)	19 (0.6)	0.318
Two-drug administration	221 (12.4)	185 (12.0)	406 (12.2)	0.689
Multiple-drug administration	1755 (98.7)	1535 (99.4)	3290 (99.0)	0.061
Standard dressing care	1556 (87.5)	1367 (88.5)	2923 (88.0)	0.394
Frequent dressing changes	153 (8.6)	88 (5.7)	241 (7.3)	0.001
Drain care	725 (40.8)	414 (26.8)	1139 (34.3)	<0.001
Mechanical ventilation	1627 (91.5)	1534 (99.3)	3161 (95.1)	<0.001
HFNC or CPAP respiratory support	561 (31.6)	121 (7.8)	682 (20.5)	<0.001
Respiratory support without mechanical ventilation	1510 (84.9)	617 (39.9)	2127 (64.0)	<0.001
Artificial airway care	1655 (93.1)	1527 (98.8)	3182 (95.8)	<0.001
Respiratory physiotherapy	1157 (65.1)	1029 (66.6)	2186 (65.8)	0.354
Single vasoactive drug	1434 (80.7)	1013 (65.6)	2447 (73.6)	<0.001
Multiple vasoactive drugs	935 (52.6)	1175 (76.1)	2110 (63.5)	<0.001
Massive fluid loss or high fluid administration	497 (28.0)	455 (29.4)	952 (28.6)	0.341
Arterial catheter	1749 (98.4)	1516 (98.1)	3265 (98.3)	0.589
Pulmonary artery catheter	6 (0.3)	3 (0.2)	9 (0.3)	0.517
Central venous catheter	1689 (95.0)	1506 (97.5)	3195 (96.1)	<0.001
Cardiopulmonary resuscitation	40 (2.2)	337 (21.8)	377 (11.3)	<0.001
Calibrated minimally invasive hemodynamic monitoring	57 (3.2)	63 (4.1)	120 (3.6)	0.179
Non-calibrated minimally invasive hemodynamic monitoring	1 (0.1)	0 (0.0)	1 (0.0)	1.000
Renal replacement therapy	213 (12.0)	520 (33.7)	733 (22.1)	<0.001
Urine output measurement	1756 (98.8)	1510 (97.7)	3266 (98.3)	0.023
Forced diuresis	1588 (89.3)	1320 (85.4)	2908 (87.5)	<0.001
Dialysis catheter insertion	155 (8.7)	396 (25.6)	551 (16.6)	<0.001
Treatment of acidosis/alkalosis	207 (11.6)	717 (46.4)	924 (27.8)	<0.001
Parenteral nutrition	1081 (60.8)	753 (48.7)	1834 (55.2)	<0.001
Enteral nutrition	1401 (78.8)	1036 (67.1)	2437 (73.3)	<0.001
Single ICU intervention	903 (50.8)	757 (49.0)	1660 (50.0)	0.303
Multiple ICU interventions	214 (12.0)	171 (11.1)	385 (11.6)	0.385
Procedures outside the ICU	771 (43.4)	570 (36.9)	1341 (40.4)	<0.001
ICP monitoring	46 (2.6)	42 (2.7)	88 (2.6)	0.814
ICU stay and admission laboratory/gas-exchange variables				
Length of ICU stay, days, mean (SD)	17.6 (28.7)	12.3 (16.2)	15.1 (23.8)	<0.001
CRP at admission, mg/L, mean (SD)	127.6 (89.3)	146.1 (109.5)	135.9 (99.3)	<0.001
WBC at admission, 10^9/L, mean (SD)	12.9 (6.6)	15.9 (16.2)	14.3 (12.0)	<0.001
Procalcitonin, ng/mL, mean (SD)	4.9 (12.1)	8.8 (16.3)	6.7 (14.2)	<0.001
Interleukin-6, pg/mL, mean (SD)	305.9 (567.0)	565.8 (857.9)	437.1 (739.2)	<0.001
PaO2, mmHg, mean (SD)	102.9 (23.8)	100.6 (31.8)	101.8 (28.0)	0.157
PaCO2, mmHg, mean (SD)	41.1 (6.8)	43.6 (10.3)	42.3 (8.8)	<0.001
pH, mean (SD)	7.43 (0.06)	7.35 (0.13)	7.39 (0.11)	<0.001
Creatinine, mg/dL, mean (SD)	1.3 (1.0)	1.9 (1.3)	1.6 (1.2)	<0.001
Glucose, mg/dL, mean (SD)	148.5 (39.8)	160.1 (56.5)	154.1 (48.9)	<0.001
Sodium, mmol/L, mean (SD)	139.8 (5.0)	140.7 (6.2)	140.2 (5.6)	0.005
Potassium, mmol/L, mean (SD)	4.0 (0.4)	4.4 (0.7)	4.2 (0.6)	<0.001
Total hemoglobin, g/dL, mean (SD)	11.3 (1.9)	11.3 (2.2)	11.3 (2.1)	0.865
Bicarbonate mmol/L, mean (SD)	26.6 (4.2)	23.6 (6.4)	25.2 (5.6)	<0.001
Lactate, mmol/L, mean (SD)	1.8 (1.3)	4.4 (5.1)	3.1 (3.9)	<0.001

Values are presented as mean (SD) or *n* (%), as appropriate. TISS-28 duration variables represent the number of ICU days on which a given intervention was recorded. Binary TISS-28 variables indicate whether the intervention was recorded at least once during the ICU stay. *p*-values were calculated using Welch *t*-tests for continuous variables and chi-square or Fisher exact tests for categorical variables.

**Table 2:** Adjusted logistic regression model for ICU mortality.

Variable	Adjusted OR	95% CI	*p*-Value
Age, per 10 years	1.32	1.18–1.47	<0.001
Female sex	1.02	0.75–1.39	0.880
Arterial hypertension	1.05	0.77–1.44	0.759
Diabetes mellitus	0.75	0.51–1.10	0.136
Ischemic heart disease	1.48	0.82–2.67	0.189
Atrial fibrillation	1.44	0.93–2.24	0.101
Renal failure	0.86	0.47–1.58	0.628
Heart and/or respiratory failure	1.01	0.70–1.47	0.956
Acute myocardial infarction or ischemic stroke	2.37	1.28–4.37	0.006
COVID-19	3.15	2.07–4.79	<0.001
Bloodstream infection	1.07	0.71–1.62	0.741
Catheter-associated urinary tract infection	1.05	0.69–1.61	0.821
Ventilator-associated pneumonia	1.68	1.22–2.30	0.001
Lactate, per 1 mmol/L	1.29	1.16–1.43	<0.001
pH, per 0.1-unit decrease	1.79	1.41–2.29	<0.001
Creatinine, per 1 mg/dL	1.08	0.92–1.26	0.366
Mechanical ventilation	14.74	3.40–63.87	<0.001
Multiple vasoactive drugs	1.40	1.04–1.90	0.027
Cardiopulmonary resuscitation	9.45	4.67–19.13	<0.001
Renal replacement therapy	2.01	1.39–2.91	<0.001
Treatment of acidosis/alkalosis	1.95	1.29–2.94	0.002

The model was fitted as a complete-case exploratory prognostic association model (*n* = 1278; deaths = 616). OR, odds ratio; CI, confidence interval.

**Table 3:** Missing data for major variables.

Variable	*n* Available	Missing, *n* (%)
Age	3262/3323	61 (1.8)
Height	2037/3323	1286 (38.7)
Weight	2033/3323	1290 (38.8)
BMI	2016/3323	1307 (39.3)
Length of ICU stay	3279/3323	44 (1.3)
Time from ICU admission to death among non-survivors	1545/1545	0 (0.0)
CRP at admission	1912/3323	1411 (42.5)
WBC at admission	2454/3323	869 (26.2)
Procalcitonin at admission	1698/3323	1625 (48.9)
Interleukin-6 at admission	408/3323	2915 (87.7)
PaO2 at admission	1286/3323	2037 (61.3)
PaCO2 at admission	1287/3323	2036 (61.3)
pH at admission	1287/3323	2036 (61.3)
Creatinine at admission	1280/3323	2043 (61.5)
Glucose at admission	1287/3323	2036 (61.3)
Sodium at admission	1287/3323	2036 (61.3)
Potassium at admission	1287/3323	2036 (61.3)
Total hemoglobin at admission	1286/3323	2037 (61.3)
Bicarbonate at admission	1287/3323	2036 (61.3)
Lactate at admission	1284/3323	2039 (61.4)
Sex, mortality outcome, infection variables, COVID-19, comorbidities, pharmacological treatment variables, and binary TISS-28 indicators	3323/3323	0 (0.0)

For time from ICU admission to death, the denominator is ICU non-survivors only. For all other rows, the denominator is the final analytic cohort (*n* = 3323).

**Table 4:** Sensitivity adjusted logistic regression model for ICU mortality after excluding COVID-19 patients.

Variable	Adjusted OR	95% CI	*p*-Value
Age, per 10 years	1.31	1.16–1.48	<0.001
Female sex	1.10	0.77–1.57	0.611
Arterial hypertension	0.87	0.60–1.27	0.477
Diabetes mellitus	0.68	0.43–1.08	0.101
Ischemic heart disease	1.47	0.73–2.93	0.280
Atrial fibrillation	1.64	1.01–2.67	0.047
Renal failure	0.70	0.34–1.41	0.313
Heart and/or respiratory failure	0.97	0.64–1.48	0.902
Acute myocardial infarction or ischemic stroke	2.72	1.47–5.04	0.001
Bloodstream infection	1.11	0.67–1.83	0.687
Catheter-associated urinary tract infection	1.20	0.74–1.96	0.466
Ventilator-associated pneumonia	1.69	1.18–2.41	0.004
Lactate, per 1 mmol/L	1.32	1.18–1.48	<0.001
pH, per 0.1-unit decrease	1.71	1.30–2.25	<0.001
Creatinine, per 1 mg/dL	1.10	0.92–1.30	0.305
Mechanical ventilation	7.39	1.65–33.15	0.009
Multiple vasoactive drugs	1.61	1.14–2.27	0.007
Cardiopulmonary resuscitation	5.78	2.73–12.22	<0.001
Renal replacement therapy	2.27	1.50–3.44	<0.001
Treatment of acidosis/alkalosis	1.49	0.94–2.35	0.087

The sensitivity model excluded COVID-19 patients and was fitted as a complete-case exploratory prognostic association model (*n* = 986; deaths = 429). OR, odds ratio; CI, confidence interval.

## Data Availability

The datasets analyzed during the current study are available from the corresponding author on reasonable request. The data are not publicly available because of institutional hospital policies and patient confidentiality requirements.

## Abbreviations

The following abbreviations are used in this manuscript:

APACHE II, Acute Physiology and Chronic Health Evaluation II; BMI, body mass index; BSI, bloodstream infection; CA-UTI, catheter-associated urinary tract infection; CIED, cardiac implantable electronic device; COVID-19, coronavirus disease 2019; CPAP, continuous positive airway pressure; CPR, cardiopulmonary resuscitation; CRP, C-reactive protein; CRT, cardiac resynchronization therapy; HFNC, high-flow nasal cannula; ICD, implantable cardioverter-defibrillator; ICU, intensive care unit; ICP, intracranial pressure; IL-6, interleukin-6; PaCO2, arterial partial pressure of carbon dioxide; PaO2, arterial partial pressure of oxygen; PCT, procalcitonin; RRT, renal replacement therapy; SAPS II, Simplified Acute Physiology Score II; SD, standard deviation; SOFA, Sequential Organ Failure Assessment; TISS-28, Therapeutic Intervention Scoring System-28; VAP, ventilator-associated pneumonia; WBC, white blood cell count; WCD, wearable cardioverter-defibrillator.
